# Gas-Selective Catalytic Regulation by a Newly Identified Globin-Coupled Sensor Phosphodiesterase Containing an HD-GYP Domain from the Human Pathogen *Vibrio fluvialis*

**DOI:** 10.1021/acs.biochem.3c00484

**Published:** 2024-01-24

**Authors:** Kenichi Kitanishi, Nao Aoyama, Motoyuki Shimonaka

**Affiliations:** † Department of Chemistry, Faculty of Science, Tokyo University of Science, 1-3 Kagurazaka, Shinjuku-ku, Tokyo 162-8601, Japan; ‡ Department of Chemistry, Graduate School of Science, Tokyo University of Science, 1-3 Kagurazaka, Shinjuku-ku, Tokyo 162-8601, Japan

## Abstract

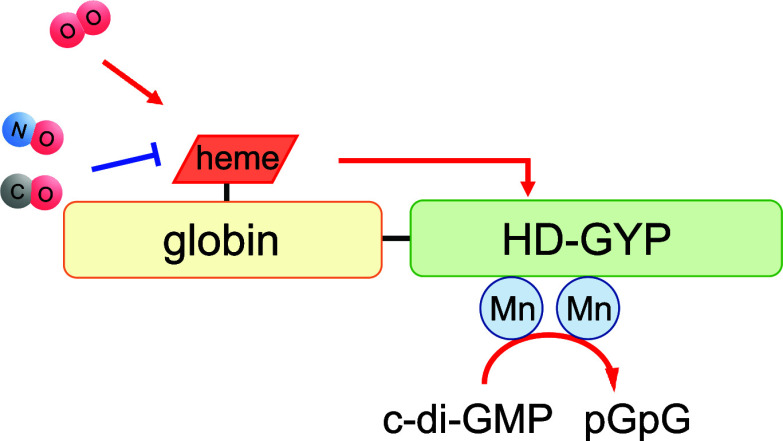

Globin-coupled sensors constitute an important family of heme-based gas sensors, an emerging class of heme proteins. In this study, we have identified and characterized a globin-coupled sensor phosphodiesterase containing an HD-GYP domain (GCS-HD-GYP) from the human pathogen *Vibrio fluvialis*, which is an emerging foodborne pathogen of increasing public health concern. The amino acid sequence encoded by the *AL536_01530* gene from *V. fluvialis* indicated the presence of an N-terminal globin domain and a C-terminal HD-GYP domain, with HD-GYP domains shown previously to display phosphodiesterase activity toward bis(3′,5′)-cyclic dimeric guanosine monophosphate (c-di-GMP), a bacterial second messenger that regulates numerous important physiological functions in bacteria, including in bacterial pathogens. Optical absorption spectral properties of GCS-HD-GYP were found to be similar to those of myoglobin and hemoglobin and of other bacterial globin-coupled sensors. The binding of O_2_ to the Fe(II) heme iron complex of GCS-HD-GYP promoted the catalysis of the hydrolysis of c-di-GMP to its linearized product, 5′-phosphoguanylyl-(3′,5′)-guanosine (pGpG), whereas CO and NO binding did not enhance the catalysis, indicating a strict discrimination of these gaseous ligands. These results shed new light on the molecular mechanism of gas-selective catalytic regulation by globin-coupled sensors, with these advances apt to lead to a better understanding of the family of globin-coupled sensors, a still growing family of heme-based gas sensors. In addition, given the importance of c-di-GMP in infection and virulence, our results suggested that GCS-HD-GYP could play an important role in the ability of *V. fluvialis* to sense O_2_ and NO in the context of host–pathogen interactions.

## Introduction

Heme, alternatively known as iron protoporphyrin IX, is one of the best-known and most important biological cofactors; it is required for many proteins and enzymes to properly perform their biological functions,^1^ for example, oxygen storage and transfer (in myoglobin and by hemoglobin, respectively), electron transfer (by cytochrome *c*), oxygen activation (by cytochrome P450 and nitric oxide synthase), and many other functions.^1−4^ These heme proteins utilize heme as a reaction center or a catalytic center. In addition to these classical heme proteins, a new class of heme proteins, namely heme-based gas sensors, which utilize heme as the gas-sensing site of various gaseous molecules including O_2_, CO, and NO, have been becoming a topic of increasing research interest in recent years.^3−5^

In general, a heme-based gas sensor protein is composed of an N-terminal heme-bound gas sensor domain and a C-terminal functional domain. Association/dissociation of the gaseous molecules with/from the heme iron triggers conformational changes in the sensor domain as an initial input signal. Then, these local conformational changes are propagated to the functional domain as a secondary signal and thereby induces global structural changes in the full-length protein, with a switching on/off of the transcription or catalytic reactions as a final output.^4−7^ Within the family of heme-based gas sensors, globin-coupled sensors make up a particularly important class and are specifically oxygen-sensor proteins in which the heme-bound sensor domain contains a globin fold similar to those of myoglobin and hemoglobin.^6,8,9^

To date, many globin-coupled sensors with various functional domains have been identified and characterized, and these sensors are responsible for regulating important bacterial physiological processes such as chemotaxis, signal transduction, and stressosome signaling.^6,8^ In addition to these functions, many globin-coupled sensors catalyzing the synthesis of the bacterial second messenger, bis(3′,5′)-cyclic dimeric guanosine monophosphate (c-di-GMP), have been identified and characterized.^6,8^ In contrast, globin-coupled sensors catalyzing the degradation of c-di-GMP have not yet been identified and characterized.

c-di-GMP is a universal second messenger that regulates a variety of processes, including cell motility, differentiation, development, antibiotic production, and biofilm formation in bacteria,^10−13^ and has also been reported to be involved in infection and virulence in some bacterial pathogens.^12,14−16^ The intracellular level of c-di-GMP is regulated specifically by the GGDEF domain of diguanylate cyclase (DGC) and the EAL or HD-GYP domain of phosphodiesterase (PDE), with the GGDEF domain catalyzing the synthesis of c-di-GMP from two molecules of GTP and the EAL or HD-GYP domain catalyzing the degradation of c-di-GMP into 5′-phosphoguanylyl-(3′,5′)-guanosine (pGpG) and/or guanosine 5′-monophosphate (GMP). The nomenclature for each domain corresponds to a subset of conserved amino acid motifs that are essential for enzymatic activity. Low and high intracellular c-di-GMP levels correlate with motile and sessile phenotypes, respectively.^10,11,17^

**Figure 1:**
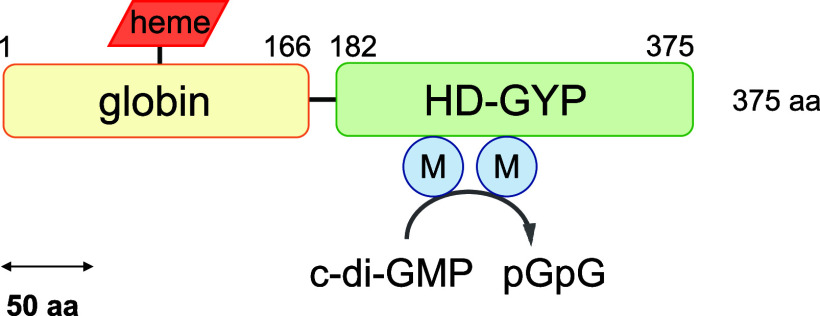
Domain architecture of GCS-HD-GYP. Heme is bound to the N-terminal globin domain, whereas the C-terminal HD-GYP domain functions as a c-di-GMP-specific PDE in the presence of divalent metals.

In this study, we identified a novel globin-coupled sensor PDE containing HD-GYP domain from the human pathogen *Vibrio fluvialis*, which is an emerging foodborne pathogen commonly found in coastal environments and which causes diarrheal outbreaks and sporadic extraintestinal issues.^18^ We identified *AL536_01530* to be the gene for this sensor, and have here designated this gene as *GCS-HD-GYP* after its domain arrangement (globin-coupled sensor and HD-GYP) (Figure 1), and characterized the spectral and catalytic properties of the full-length protein. Purified GCS-HD-GYP was clearly found to be a dimeric heme-bound protein with spectral properties similar to those of myoglobin and hemoglobin. Significant PDE activities toward c-di-GMP were observed for the Fe(III) and Fe(II)-O_2_ forms of GCS-HD-GYP but not for the Fe(II), Fe(II)-CO, and Fe(II)-NO forms, indicating strict discrimination of these gaseous ligands. These results suggested the importance of GCS-HD-GYP for sensing O_2_ and NO in the context of host–pathogen interactions.

## Materials and Methods

### Materials

c-di-GMP and diethylamine (DEA) NONOate were purchased from Cayman Chemical (Ann Arbor, MI). GMP and pGpG were purchased from Sigma-Aldrich (St. Louis, MO) and BIOLOG Life Science Institute (Bremen, Germany), respectively. All other chemicals were acquired from Kanto Chemical (Tokyo, Japan), Nacalai Tesque (Kyoto, Japan), or FUJIFILM Wako Pure Chemical Corporation (Osaka, Japan), were of the highest guaranteed grade available, and were used without further purification.

### Construction of Expression Plasmids

The genes encoding *Vibrio fluvialis* GCS-HD-GYP (*AL536_01530*) and *Vibrio furnissii* GCS-HD-GYP (*AMR76_07425*) were synthesized by Gene Universal (Newark, DE), and were codon-optimized for expression in *Escherichia coli*. The corresponding cDNA in each of these two cases was inserted into the pET-21a vector (Novagen, Darmstadt, Germany) using NdeI and XhoI restriction sites and included sequence for a His_6_ tag at the C terminus of the desired protein.

### Expression and Purification of GCS-HD-GYP

*E. coli* BL21(DE3) (Novagen) was transformed with a pET-21a vector expressing GCS-HD-GYP and grown overnight at 37 °C in 5 mL of Luria–Bertani medium (BD Difco) containing ampicillin (100 mg/L). Then, 1 L of the same medium containing ampicillin was inoculated with the starter culture (1:200 dilution) and grown at 37 °C. After 3 h, when the OD_600_ had reached 0.6–0.8, the temperature was reduced to 15 °C, and the protein expression was induced with 0.1 mM isopropyl β-D-thiogalactopyranoside. In the cases of adding Fe^2+^ or Mn^2+^ ions upon induction, the concentrations added were specifically 100 mg/L (NH_4_)_2_Fe(SO_4_)_2_·6H_2_O for Fe^2+^ and 100 mg/L MnCl_2_·4H_2_O for Mn^2+^. After 20 h, cells from the resulting culture were harvested by subjecting the culture to centrifugation, and the resulting cell pellets were stored at −80 °C until purification. The cell pellets (∼5 g from 1 L of culture) were suspended in 80 mL of Buffer A (50 mM Tris–HCl, pH 8.0, 100 mM NaCl) containing 1 mM phenylmethanesulfonyl fluoride. The cell suspension was stirred at 4 °C for 30 min and then sonicated (power setting, 5; duty, 50) on ice for a total of 6 min, with specifically three 2 min intervals (separated by 2 min cooling periods), using an ultrasonic disrupter (UD-201; TOMY SEIKO, Tokyo, Japan). The sonicate was centrifuged at 35,870*g* for 30 min, and the resulting supernatant was incubated for 5 min with 50 μM hemin chloride in a dimethyl sulfoxide solution, and then loaded onto a HisTrap HP column (Cytiva) pre-equilibrated with Buffer A containing 20 mM imidazole. The column was washed with 150 mL of Buffer A containing 20 mM imidazole and eluted with 100 mL of a linear gradient of imidazole, specifically from 20 to 300 mM imidazole, in Buffer A. The fractions of interest in the resulting eluate were pooled and dialyzed overnight against 1 L of Buffer A. The dialyzed protein was concentrated to that in a volume of 5 mL using an Amicon Ultra-15 centrifugal filter device (Merck Millipore) and loaded onto a HiLoad 16/600 Superdex 200 pg column (Cytiva) pre-equilibrated with Buffer A. The fractions of interest in the resulting eluate were pooled, concentrated, frozen in liquid nitrogen, and stored at −80 °C until further use. Protein concentrations were determined by using the Bradford protein assay with bovine serum albumin as a standard, and heme concentrations were determined using the pyridine hemochromogen method. GCS-HD-GYP was found to bind heme at a 1:1 molar ratio, so its protein concentration is expressed below in terms of heme concentration, with this relationship between protein concentration and heme concentration further described below.

### Metal Content

Metal content analysis was performed by carrying out inductively coupled plasma optical emission spectroscopy (ICP-OES) using a SPECTRO ARCOS FHM22 apparatus (SPECTRO Analytical Instruments, Kleve, Germany). The metal content was determined based on transitions of the atomic emission spectrum. Solutions of a protein sample and reference standard in 0.1 M nitric acid were prepared.

### Size-Exclusion Chromatography Analysis

To determine the oligomerization state, size-exclusion chromatography was carried out using an ÄKTAprime plus (GE Healthcare) chromatography system equipped with a Superdex 200 Increase 10/300 GL column (GE Healthcare). The buffer used for this chromatography was 50 mM Tris–HCl, pH 8.0, and 100 mM NaCl. For each sample, 100 μL of a solution of 20 μM protein was injected. The molecular weight was estimated from the correlation between the molecular weight and elution volume of standard proteins using a gel filtration molecular weight marker kit (Sigma-Aldrich).

### Far-UV Circular Dichroism (CD) Spectra

Far-UV CD spectra were recorded with a JASCO J-820 CD spectropolarimeter (Tokyo, Japan) using a demountable rectangular quartz cell (0.1 mm path length). The spectral data were collected four times at a bandwidth of 1 nm, scan speed of 20 nm/min, and response time of 4 s, and then, the data were combined. A protein concentration of 20 μM and buffer consisting of 20 mM Tris–HCl, pH 8.0, and 100 mM NaCl were used. The contents of α-helix, β-sheet, and turns were estimated by using JWSSE-480 (JASCO) software with a classical least-squares method using reference data by Yang et al.^19^

### Optical Absorption Spectra

Absorption spectral data were obtained using a V-630BIO or V-750 spectrophotometer (JASCO). Gas-saturated solutions were obtained by bubbling buffers (50 mM Tris–HCl, pH 8.0, 100 mM NaCl) with the appropriate gas for at least 30 min at room temperature. The Fe(II) complex was prepared in a N_2_-saturated buffer by adding sodium dithionite to the Fe(III) complex. The Fe(II)-O_2_ complex was prepared by reducing the Fe(III) complex with 10 mM sodium dithionite; and then the resulting mixture was applied to a Micro Bio-Spin 6 column (Bio-Rad Laboratories) to carry out desalting, specifically to remove excess dithionite. The Fe(II)-CO complex was prepared in a CO-saturated buffer by reducing the Fe(III) complex with 10 mM sodium dithionite. The Fe(II)-NO complex was prepared by adding 0.1–0.2 mM of the NO donor DEA NONOate to the solution of the Fe(II) complex.

### Dependence of Absorption Spectral Data on pH

Absorption spectra of samples of different pH values were acquired by using a JASCO V-750 spectrophotometer. The pH buffers used were 50 mM sodium phosphate buffer (pH 6.0, 6.5, and 7.0), 50 mM Tris–HCl buffer (pH 8.0, and 9.0), and 50 mM sodium carbonate buffer (pH 10.0, and 11.0).

### Enzymatic Assays

PDE activity was typically assayed at 20 °C in a reaction mixture containing 50 mM Tris–HCl, pH 8.0, 100 mM NaCl, 1 mM MnCl_2_, and 1 μM GCS-HD-GYP unless otherwise stated. The reaction mixture was preincubated for 5 min, and the reaction was initiated by adding 0.1 mM c-di-GMP to the mixture. At the indicated times, the reaction was stopped by incubating the mixture for 5 min at 95 °C, followed by subjecting it to centrifugation for 10 min at 16,000*g* to remove any precipitate. The supernatant samples (10 μL) were injected into a LUNA 5 μm C18 (2) column (150 mm × 4.6 mm; Phenomenex, Torrance, CA) using an autosampler (AS-2057 Plus, JASCO) and analyzed using an HPLC system consisting of a gradient pump (PU-2089 Plus, JASCO) and a UV/vis detector (UV-2075 Plus, JASCO). Nucleotides in these injected samples were detected by measuring their absorbance at 254 nm. Chromatographic data were acquired by using a Chromato-PRO system (Run Time Instruments, Tokyo). The solvents used in the gradient program were Solvent A (0.1 M KH_2_PO_4_ with 4 mM tetrabutylammonium hydrogen sulfate (pH 6.0)) and Solvent B (75% Solvent A/25% methanol). The gradient was delivered at a flow rate of 0.7 mL/min according to the following program: 0 min, 40% B/60% A; 15 min, 100% B; 20 min, 100% B; 21 min, 40% B/60% A. Retention times were 5.79 min for GMP, 14.2 min for pGpG, and 18.3 min for c-di-GMP. Every experiment was conducted in triplicate.

## Results

### Identification of a Novel Sensor PDE in the *V. fluvialis* Genome

Gene analysis of *AL536_01530* in the *V. fluvialis* genome revealed the presence of a globin domain at the N terminus and an HD-GYP domain at the C terminus (Figure 1). Comparison of the sequence of this globin domain with those of other globin proteins—for example, myoglobin, hemoglobin, and other bacterial globin-coupled sensors including *Bacillus subtilis* HemAT, *E. coli* YddV, and *Anaeromyxobacter* sp. Fw109-5 GcHK (*Af*GcHK) (Figure S1)—suggested that it too can bind heme. In particular, both Tyr49 at the heme distal side, and His103 at the heme proximal side were observed to be conserved among the sequences of this family (Figure S1), and are important for oxygen binding and heme binding, respectively.^20−22^ The globin domain (residues 1–166) of GCS-HD-GYP was found to be homologous to those of *B. subtilis* HemAT (residues 22–185) (19.7% identity and 41.0% similarity) and sperm whale myoglobin (12.9% identity and 24.1% similarity).

The HD-GYP domain functions as a c-di-GMP-specific PDE, which usually requires two or three divalent metals (Fe or Mn) for catalysis.^23−27^ Comparison of the relevant HD-GYP-domain-containing proteins that have been characterized recently^28−30^ with GCS-HD-GYP showed the conserved putative metal-binding residues to all be present in the GCS-HD-GYP sequence (Figure S2). In addition, the Rxx(R/K) motif—which recognizes the guanine base of c-di-GMP and is important for being specific for this substrate rather than other cyclic dinucleotides^31^—was also present in the GCS-HD-GYP sequence (corresponding to Arg319 and Lys322 in GCS-HD-GYP) (Figure S2). Our sequence comparisons with the structurally characterized HD-GYP-domain-containing PDEs showed the HD-GYP domain (residues 169–366) of GCS-HD-GYP to be homologous to that of *Bdellovibrio bacteriovorus* Bd1817 (residues 117–308) (19.9% identity and 39.3% similarity), which has been shown to contain a dinuclear iron center at its active site,^28^ and to that of *Persephonella marina* PmGH (residues 161–363) (43.8% identity and 67.5% similarity), which has been shown to contain a trinuclear iron center at its active site.^29^

Thus, we hypothesized that GCS-HD-GYP from *V. fluvialis* is a heme-bound, globin-coupled sensor enzyme with PDE activity toward c-di-GMP, and its catalytic activity could be affected by a change in the redox state of the heme iron or binding of gaseous ligand to the heme iron.

### Initial Characterization of Purified GCS-HD-GYP

When GCS-HD-GYP was expressed in *E. coli* with a supplement of the heme precursor 5-aminolevulinic acid, the expressed protein showed a dark greenish color rather than a reddish brown, indicating that heme was degraded during protein expression. This different color was also observed for other globin-coupled sensor DGCs, *Desulfotalea psychrophila* HemDGC^32^ and Leu65 mutants of *E. coli* YddV,^33^ suggesting the residue at this position could have effects on degradation of heme during protein expression in *E. coli*. Accordingly, we reconstituted GCS-HD-GYP with heme by deploying the method previously used for another globin-coupled sensor histidine kinase, namely *Af*GcHK.^21,34^ Briefly, GCS-HD-GYP was expressed in its apo (heme-free) form in *E. coli* cells. Then the cells were disrupted by subjecting them to sonication to produce a crude extract. GCS-HD-GYP was reconstituted with heme by adding heme to the crude extract and finally purified in its heme-bound form.

**Figure 2:**
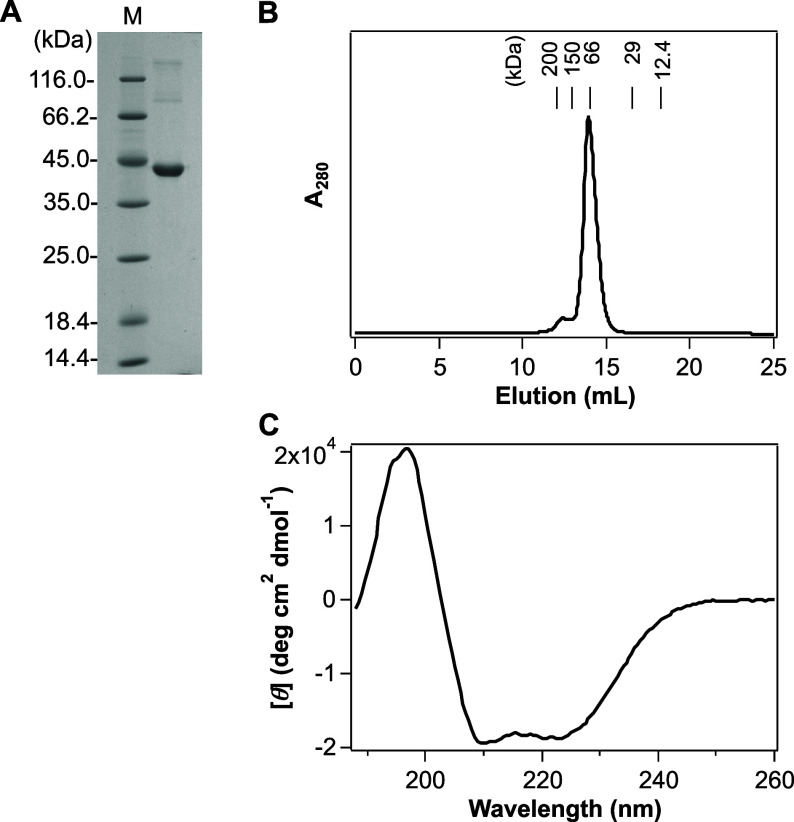
Initial characterization of GCS-HD-GYP. (A) Purity of GCS-HD-GYP was judged based on a 12% SDS-PAGE analysis. Molecular mass markers were run on the left lane. (B) Elution profile of a GCS-HD-GYP sample from a size-exclusion column, revealing that it behaved as a dimeric protein. The molecular masses of standard proteins are shown at the top. (C) Far-UV CD spectrum of GCS-HD-GYP at a concentration of 20 μM in a buffer consisting of 20 mM Tris–HCl, pH 8.0, 100 mM NaCl.

This purified GCS-HD-GYP contained 1 equiv of heme iron, as confirmed using the pyridine hemochromogen method and ICP-OES, as discussed later. The purity of the full-length GCS-HD-GYP protein was judged to be >90% according to an SDS-PAGE analysis, and the band observed in the SDS-PAGE gel corresponded to a predicted mass of 44.1 kDa for the full-length protein with a C-terminal His_6_ tag (Figure 2A). Size-exclusion chromatography analysis showed a main elution peak (14.0 mL) and a minor elution peak (∼12.3 mL), which were calculated to correspond to molecular masses of 85 and 176 kDa, respectively (Figure 2B), and hence corresponded to dimer and tetramer, respectively. This result clearly indicated that GCS-HD-GYP would in general form mostly a dimer in solution.

To investigate the secondary structure of the purified GCS-HD-GYP protein, we acquired its far-UV CD spectrum. The spectrum exhibited minima at 210 and 223 nm (Figure 2C), indicative of a primarily helical structure. The α-helix content of GCS-HD-GYP was estimated to be 50–60%, and the remainder of the protein formed turns and random structures without any β-sheets. Based on the structural model of the full-length protein predicted using AlphaFold (Figure S3),^35,36^ the globin domain was predicted to be a helical protein, specifically consisting of at least 8 helices. Similarly, the HD-GYP domain also consisted of at least 9 helices. Hence, the full-length protein was concluded to consist of mainly α-helices along with the turns connecting them.

### Optical Absorption Spectra of Purified GCS-HD-GYP

**Figure 3:**
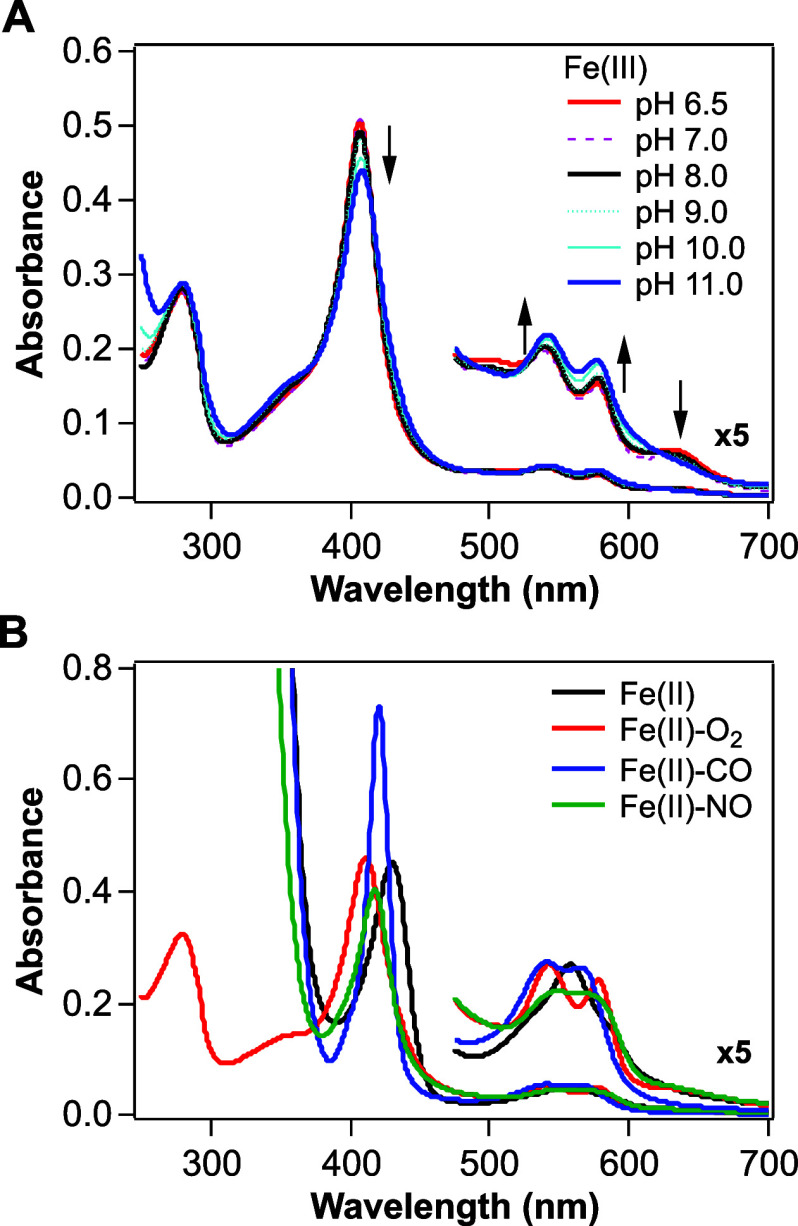
Absorption spectra of various complexes of GCS-HD-GYP. The protein concentration used in these experiments was 4 μM. The inset in each panel shows a 5-fold *y*-axis enlargement of the visible region of the spectrum (475–700 nm). The absorption maxima of these proteins are given in Table 1. (A) Spectra of the Fe(III) complex of GCS-HD-GYP at indicated pH levels. The buffers used in these experiments are described in Materials and Methods. (B) Spectra of the Fe(II), Fe(II)-O_2_, Fe(II)-CO, and Fe(II)-NO complexes of GCS-HD-GYP. The buffer used in these experiments was 50 mM Tris–HCl, pH 8.0, and 100 mM NaCl.

Heme proteins display characteristic spectral features, especially the Soret band (at about 400 nm) and visible bands between 500 and 700 nm, attributed to the π–π* transition of the porphyrin ring and utilized as a fingerprint to understand the heme coordination structure. Optical absorption spectra of GCS-HD-GYP in its oxidized (Fe(III)), reduced (Fe(II)), and gas-bound (Fe(II)-O_2_, Fe(II)-CO, and Fe(II)-NO) forms were acquired (Figure 3). The absorption maxima of these spectra and those of other relevant globin-coupled sensors and myoglobin are given in Table 1.

**Table 1:** Absorption Maxima (nm) of the Fe(III), Fe(II), Fe(II)-O_2_, Fe(II)-CO, and Fe(II)-NO Complexes of *V. fluvialis* and *V. furnissii* GCS-HD-GYPs^a^

	Fe(III)	Fe(II)	Fe(II)-O_2_	Fe(II)-CO	Fe(II)-NO
GCS-HD-GYP					
*V. fluvialis*	407, 497, 541, 578, 633	430, 558	411, 542, 578	421, 540, 566	418, 549, 568
	His/H_2_O or OH^–^	His	His/O_2_	His/CO	His/NO
	6cHS, 6cLS	5cHS	6cLS	6cLS	6cLS
*V. furnissii*	408, 533, 573, 634	428, 559	410, 539, 575	420, 539, 567	416, 546, 570
	His/H_2_O or OH^–^	His	His/O_2_	His/CO	His/NO
	6cHS, 6cLS	5cHS	6cLS	6cLS	6cLS
YddV^b^	394, 506, 651	432, 560	413, 542, 578	420, 539, 566	417, 543, 572
	His	His	His/O_2_	His/CO	His/NO
	5cHS	5cHS	6cLS	6cLS	6cLS
*Af*GcHK^c^	411, 538	431, 559	413, 545, 580	420, 541, 565	not reported
	His/OH^–^	His	His/O_2_	His/CO	
	6cLS	5cHS	6cLS	6cLS	
SwMb^d^	410, 505, 635^e^	434, 556	418, 543, 581	423, 542, 579	420, 548, 579
	His/H_2_O	His	His/O_2_	His/CO	His/NO
	6cHS	5cHS	6cLS	6cLS	6cLS
	414, 542, 582^e^				
	His/OH^–^				
	6cLS				

a Corresponding maxima from the spectra of other relevant globin-coupled sensors and sperm whale myoglobin (SwMb) are shown as references. Proposed coordination structures are also presented: 6cHS, 6-coordinated high-spin; 6cLS, 6-coordinated low-spin; and 5cHS, 5-coordinated high-spin.

b Ref (20).

c Ref (21).

d Refs (2 and 37).

e For comparison with GCS-HD-GYP, both acid (H_2_O) and alkaline (OH^–^) forms are shown.

The Fe(III) complex of purified GCS-HD-GYP at pH 8.0 exhibited a Soret band at 407 nm and several bands at visible regions of 497, 541, 578, and 633 nm (Figure 3A, black bold line and Table 1). Especially note the charge transfer band at ∼630 nm, characteristic of a 6-coordinated high-spin (6cHS) complex with H_2_O as a sixth ligand (Figure 3A and Table 1).^2^ Moreover, based on a comparison with the absorption spectra of acidic (H_2_O) and alkaline (OH^–^) forms of myoglobin (Table 1),^2^ this spectrum was assignable to a mixture of 6cHS and 6-coordinated low-spin (6cLS) states, and H_2_O and OH^–^ were each suggested to be the sixth ligand trans to the fifth axial ligand, His103.

To further confirm the heme coordination structure of the Fe(III) complex of GCS-HD-GYP, the dependence of its absorption spectrum on pH was studied (Figure 3A). Although precipitates formed below pH 6.0 (calculated pI = 5.95), a decrease in the intensity of the Soret band with a slight shift of its peak from 407 to 408 nm and a concomitant increase in the intensities of the α (∼578 nm) and β (∼541 nm) bands were observed as the pH was increased from 6.5 to 11.0. The charge transfer band at ∼630 nm completely disappeared at pH 11.0 (Figure 3A), indicative of a complete conversion to a typical 6cLS complex with OH^–^ as a heme axial ligand. Accordingly, the axial ligand of the Fe(III) complex of GCS-HD-GYP was suggested to be in equilibrium between H_2_O and OH^–^ at neutral pH, and converted to OH^–^ at the highly alkaline pH. These results also suggested an estimated value of between 8 and 9 for this “acid-alkaline transition” equilibrium constant (p*K*_a_) for GCS-HD-GYP, whereas the corresponding values for sperm whale myoglobin and human hemoglobin have been measured to be 8.99 and 8.05, respectively.^2^

The Fe(II) complex was formed by adding excess sodium dithionite to the Fe(III) complex and showed the Soret peak at 430 nm and a single peak at 558 nm in the visible region (Figure 3B and Table 1). Diatomic gaseous ligands O_2_, CO, and NO can bind to the Fe(II) complex, and Soret absorption maxima of the Fe(II)-O_2_, Fe(II)-CO, and Fe(II)-NO complexes were observed at 411, 421, and 418 nm, respectively (Figure 3B and Table 1). The Fe(II) complex was a 5-coordinated high-spin (5cHS) complex, whereas Fe(II)-O_2_, Fe(II)-CO, and Fe(II)-NO complexes were 6cLS complexes (Table 1). All of the complexes of GCS-HD-GYP except for the Fe(III) complex showed spectral properties similar to those previously reported for other bacterial globin-coupled sensors including *E. coli* YddV and *Af*GcHK, and sperm whale myoglobin (Table 1).^2,20,21,37^ The unique property of the Fe(III) complex of GCS-HD-GYP is likely due to the structure surrounding the heme. Further studies using other spectroscopic methods, such as resonance Raman, electron paramagnetic resonance (EPR), and magnetic circular dichroism (MCD) are required to confirm this unique property.

### Metal Content of Purified GCS-HD-GYP

HD-GYP domains usually require two or three divalent metal ions for catalysis,^26^ and these metals are in some cases already bound to the active site in the HD-GYP domain of as-purified proteins. We attempted to identify and quantify any metals bound in GCS-HD-GYP. ICP-OES analysis showed that the as-purified GCS-HD-GYP contained only 1 equiv of iron atoms per monomer (Table S1), indicating binding of 1 equiv of heme iron to this as-purified protein at the N-terminal globin domain; hardly any or absolutely no iron was bound at the C-terminal HD-GYP domain, as confirmed using the pyridine hemochromogen method, as described above; and the ICP-OES analysis showed no other metal besides iron having been detected (Table S1), indicating a lack of any metals bound to the C-terminal HD-GYP domain of the as-purified protein, probably due to the low metal affinity.

Previous studies on other HD-GYP-domain-containing PDEs, namely, *Thermotoga maritima* TM0186 and *Ferrovum* sp. PN-J185 Bhr-HD-GYP, demonstrated that the protein expression in *E. coli* cells supplemented with divalent metals (e.g., Fe^2+^ or Mn^2+^) produced proteins containing metals at the active site in the HD-GYP domain.^24,38^ However, in the case of GCS-HD-GYP in our current study, even the addition of Fe^2+^ (in the form of (NH_4_)_2_Fe(SO_4_)_2_·6H_2_O) or Mn^2+^ (in the form of MnCl_2_·4H_2_O) into the growth media during protein expression did not yield these metal-containing GCS-HD-GYP proteins, as confirmed using ICP-OES (data not shown).

### Optimization of Reaction Conditions for c-di-GMP Hydrolysis Catalyzed by Purified GCS-HD-GYP

**Figure 4:**
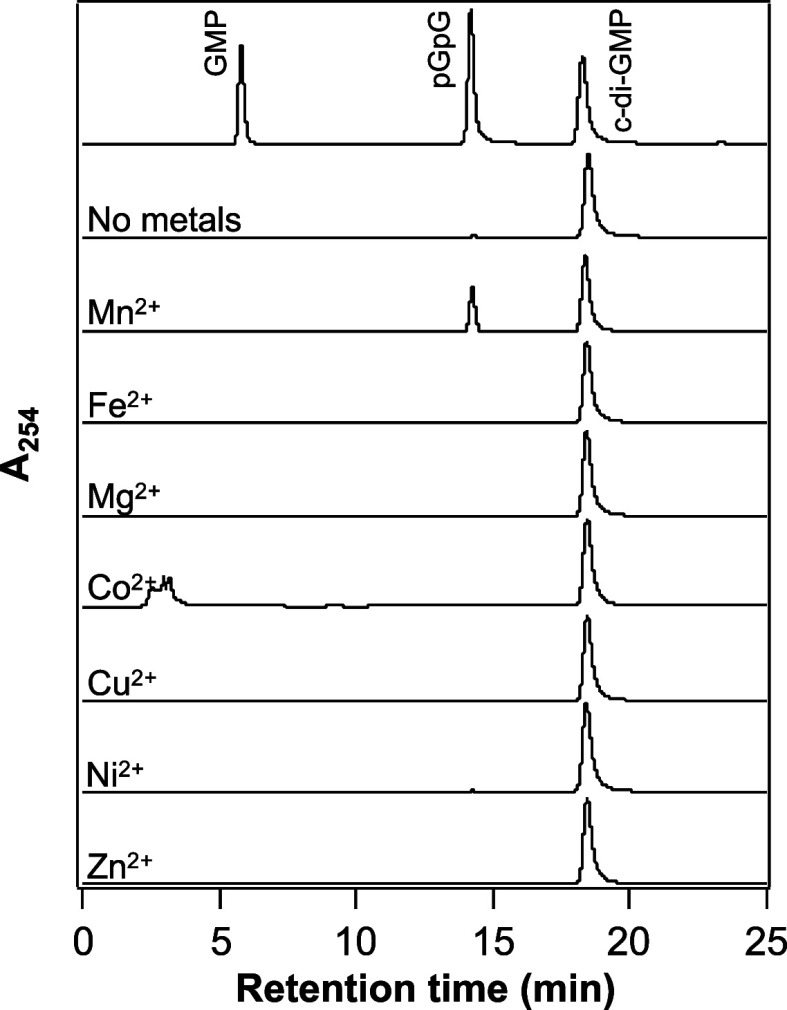
Effects of various divalent metals on c-di-GMP hydrolysis catalyzed by GCS-HD-GYP. Shown are HPLC profiles of a mixture of c-di-GMP, pGpG, and GMP standards each at a concentration of 0.1 mM (top row) and of reaction mixtures, each after 15 min of incubation at 20 °C, of 1 μM of the Fe(III) complex of GCS-HD-GYP in the presence of no metal (second row) or 1 mM MnCl_2_, FeCl_2_, MgCl_2_, CoCl_2_, CuCl_2_, NiCl_2_, or ZnCl_2_ (remaining rows). To avoid the oxidation of Fe^2+^, the assay buffer was bubbled with N_2_ gas and the FeCl_2_ solution was freshly prepared before the assay.

Due to the ICP-OES study having revealed a lack of any metals at the active site of GCS-HD-GYP despite the presence of all the putative metal-binding residues in the GCS-HD-GYP sequence (Figure S2), we set out to conduct a variety of further experiments—and first examined the effects of a range of divalent metals (1 mM Mn^2+^, Fe^2+^, Mg^2+^, Co^2+^, Cu^2+^, Ni^2+^, and Zn^2+^) on the PDE activity of the Fe(III) complex of GCS-HD-GYP toward c-di-GMP, and analyzed the reaction products using HPLC (Figure 4). In the absence of any metals, the Fe(III) complex of GCS-HD-GYP did not exhibit significant activity (Figure 4). In contrast, in the presence of Mn^2+^, a significant time-dependent decrease in the intensity of the c-di-GMP peak was observed, at 18.3 min, and was accompanied by the appearance of a new peak at 14.2 min, indicating conversion of c-di-GMP to a new compound, namely its linearized product pGpG (Figure 4). Of the Mn^2+^ concentrations tested, the catalytic activity of the Fe(III) complex of GCS-HD-GYP was highest in the presence of 1 mM Mn^2+^ (Figures 4 and S4A). Also, the protein showed almost no significant activity in the presence of any other metals, except for Mn^2+^. Thus, in subsequent experiments, we measured the catalytic activity in the presence of 1 mM Mn^2+^.

Next, we further optimized the reaction conditions for c-di-GMP hydrolysis in the presence of 1 mM MnCl_2_. We specifically optimized the reaction temperature and pH (Figure S4B,C). In our assay conditions, of the temperatures that were tested, i.e., between 10 and 35 °C, the optimum one for the catalysis of c-di-GMP hydrolysis by the Fe(III) complex of GCS-HD-GYP was 20 °C (Figure S4B); coinciding with these activity data results, *V. fluvialis* has been reported to survive in water temperatures between 9 and 31 °C but to thrive above 18 °C.^39^ Regarding pH, the Fe(III) complex of GCS-HD-GYP showed no significant activity below pH 7.0 and at pH 11.0, but showed significant activity from pH 8.0 to pH 10.0 with its activity increasing with increasing pH in this range (Figure S4C), with these results suggesting the presence of a relationship between heme coordination structure and enzymatic activity as discussed in more detail below; note that c-di-GMP itself was determined to be stable at this tested pH 6.5–11.0 region in control experiments (Figure S4D). Accordingly, all further experiments were carried out in the presence of 1 mM MnCl_2_ at pH 8.0 and 20 °C.

### Effects of Redox Change and Gas Binding on Catalysis of Purified GCS-HD-GYP

**Figure 5:**
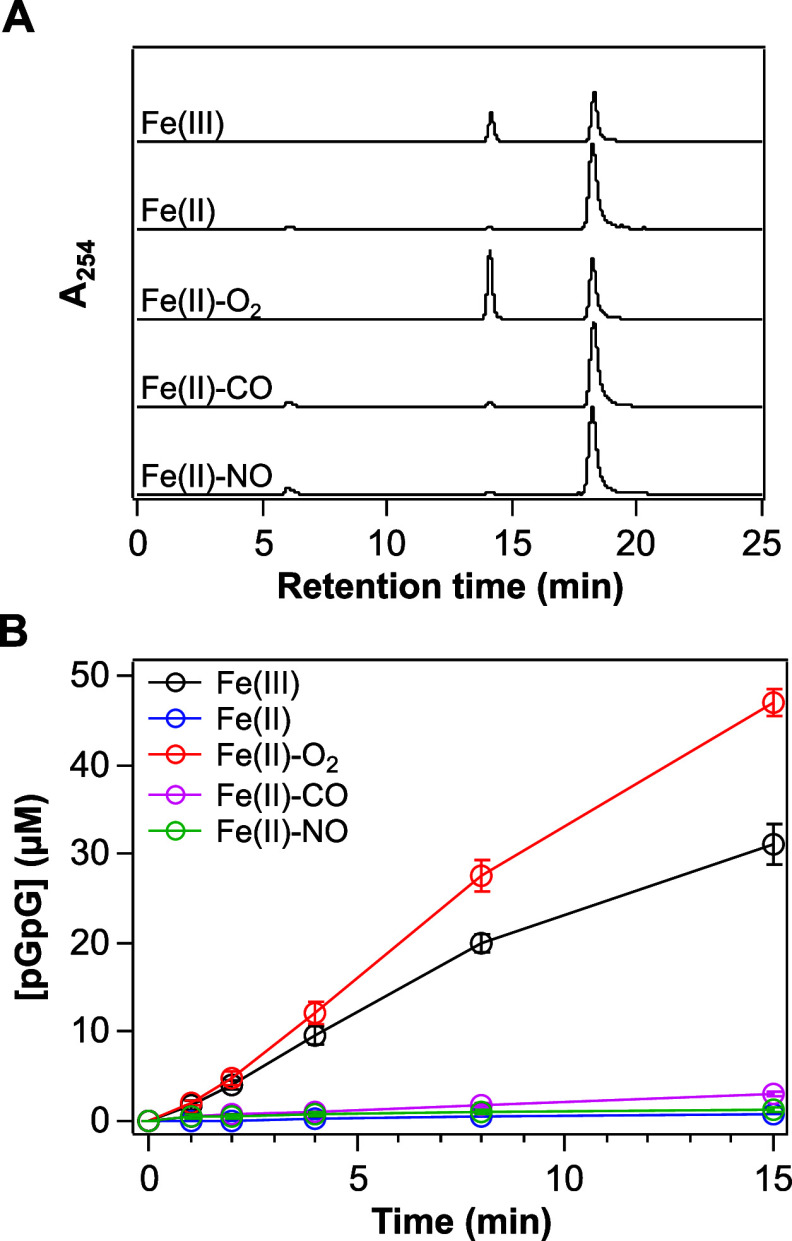
Effects of redox change and gas-binding on the PDE activity of GCS-HD-GYP. (A) HPLC chromatograms of reaction mixtures of 1 μM of indicated complexes of GCS-HD-GYP in the presence of 1 mM MnCl_2_, each after 15 min of incubation at 20 °C. Incubation of the Fe(III) and Fe(II)-O_2_ complexes of GCS-HD-GYP with c-di-GMP led to its partial hydrolysis to pGpG after 15 min, but the Fe(II), Fe(II)-CO, and Fe(II)-NO complexes of GCS-HD-GYP did not. (B) Time courses for PDE activities of the Fe(III) (black circles), Fe(II) (blue circles), Fe(II)-O_2_ (red circles), Fe(II)-CO (magenta circles), and Fe(II)-NO (green circles) complexes of GCS-HD-GYP. Each data point represents the mean ± SD of the results of at least three independent experiments. It should be noted that the autoxidation rate constant of the Fe(II)-O_2_ complex of GCS-HD-GYP was measured to be 0.028 min^–1^ at room temperature, and its half-life was 25 min, which did not affect the initial velocity; however, a later decrease in the activity, specifically after 8 min, was observed and could have been due to autoxidation.

Finally, to determine the effects of a change in the redox state of the heme and of binding of gas to the heme on enzymatic activity, we examined the PDE activities toward c-di-GMP of various heme iron complexes of GCS-HD-GYP (Figure 5). The Fe(III) and Fe(II)-O_2_ complexes of GCS-HD-GYP catalyzed the hydrolysis of c-di-GMP to form pGpG (Figure 5A). The specific activities for PDE activity displayed by the Fe(III) and Fe(II)-O_2_ complexes were, respectively, 2.6 ± 0.1 and 3.5 ± 0.2 μmol of c-di-GMP/min/(μmol of enzyme) (Figure 5B). In contrast, the Fe(II), Fe(II)-CO, and Fe(II)-NO complexes of GCS-HD-GYP did not exhibit any considerable such activity, with specific activities of only 0.06 ± 0.01, 0.20 ± 0.02, and 0.07 ± 0.01 μmol of c-di-GMP/min/(μmol of enzyme), respectively (Figure 5). Taking these results together, the order of the catalytic activities of the complexes of GCS-HD-GYP was Fe(II)-O_2_ > Fe(III) ≫ Fe(II)-CO > Fe(II) and Fe(II)-NO (Figure 5B). This trend of oxygen-dependent catalytic regulation was found to be similar to that observed for other globin-coupled sensors, including *Bordetella pertussis* GReg (*Bpe*GReg), YddV, and *Af*GcHK (i.e., with Fe(II)-O_2_ being an active form and Fe(II) an inactive form), and our results indicated the ability of GCS-HD-GYP to strictly discriminate the types of gaseous ligands, i.e., O_2_ versus CO and NO.

## Discussion

Since the discovery two decades ago of HemAT,^40^ the first member of the family of globin-coupled sensors, this family has been expanding. Many bacterial globin-coupled sensors have been identified, but none with PDE. Although a globin-coupled sensor catalyzing both the synthesis and degradation of c-di-GMP was characterized very recently,^41^ details of the catalysis of the degradation of c-di-GMP by a globin-coupled sensor have been lacking until our current study. In this study, we identified GCS-HD-GYP from *V. fluvialis* and examined its spectral and catalytic properties.

### Characteristic Features of Gas-Selective Catalytic Regulation by Globin-Coupled Sensors

For most globin-coupled sensors reported to date, catalytic reactions are markedly enhanced by the binding of O_2_ to the Fe(II) heme complex in the globin domain.^6,8^ That is, the Fe(II)-O_2_ complex is the active form, whereas the Fe(II) complex is the inactive form. However, the effects of gaseous ligands other than O_2_ on catalysis have been found to differ for different globin-coupled sensors. In GCS-HD-GYP, like HemDGC,^32^ only O_2_—and neither CO nor NO—activates catalysis, indicating the strict ligand discrimination of these sensors. In contrast, in *Bpe*GReg,^42^ CO and NO each also activate catalysis to a sufficient degree, albeit less so than does O_2_. And in YddV and *Af*GcHK, CO activates catalysis to the same degree as does O_2_.^20,21^ Furthermore, another example of gas-selective catalytic regulation was demonstrated in the recently characterized DcpG from *Paenibacillus dendritiformis*, a globin-coupled sensor displaying dual enzyme activity (DGC + PDE) and containing GGDEF and EAL domains.^41^ In DcpG, the Fe(II)-O_2_ form is, via its EAL domain, active in the degradation of c-di-GMP (PDE activity), whereas the Fe(II)-NO form is, via its GGDEF domain, active in the synthesis of c-di-GMP (DGC activity). Thus, these gaseous ligands have opposite effects on the catalysis displayed by DcpG.

### Structural Insight into Ligand Discrimination and Catalytic Regulation

Our previous studies on other globin-coupled sensors suggested that the heme coordination structure regulates the enzymatic activity of globin-coupled sensors (Figure S5).^20−22,34^ Our current study also indicated that the 6cLS (Fe(II)-O_2_) complex is active, whereas 5cHS (Fe(II)) is inactive. The Fe(II)-CO and Fe(II)-NO complexes are also 6cLS, but these complexes are inactive forms, in contrast to the Fe(II)-O_2_ complex in the case of GCS-HD-GYP, implying the presence of another factor in addition to the heme coordination structure for regulating the catalytic activity. It has been suggested that a distal Tyr residue is important for oxygen recognition, in particular, that this distal Tyr interacts using hydrogen bonds with an O_2_ molecule bound to the heme iron for the other globin-coupled sensors, but does not interact with CO (Figure S5).^20,21^ It is currently unknown whether the distal Tyr interacts with NO in globin-coupled sensors including GCS-HD-GYP. Elucidation of the binding mode of these gaseous molecules is necessary to provide a more detailed mechanism for the recognition of gaseous ligands by GCS-HD-GYP and to explain its gas selectivity. Furthermore, according to our experiments testing the effects of pH on the Fe(III) complex, the relative amount of the 6cLS (His/OH^–^) species apparently increased with increasing pH (Figure 3A), as did concomitantly the enzymatic activity (Figure S4C), indicating that the 6cLS (His/OH^–^) Fe(III) complex rather than the 6cHS (His/H_2_O) Fe(III) complex is the active form. These results further corroborated that the coordination structure of heme in the globin domain regulates its catalytic activity in the functional domain, as also demonstrated for other globin-coupled sensors.^20−22,34^

Because no structure of any full-length globin-coupled sensor has been determined to date, the mechanism underlying how signaling is transduced from the sensor domain to the functional domain is currently unknown. Recent structural models of globin-coupled sensors predicted using AlphaFold revealed the presence of one long helix connecting the globin domain and the functional domain in many globin-coupled sensors, including HemAT, YddV, and *Af*GcHK, as well as GCS-HD-GYP (Figure S3), suggesting that this putative “signaling helix” could be a common module for transducing the signal from the sensor domain to the functional domain in bacterial globin-coupled sensors. A structural comparison of the liganded (Fe(III)-CN) and unliganded (Fe(II)) complexes of the isolated globin domain of HemAT revealed a ligand-dependent conformational change to a more symmetrical state within the globin dimer and small rotational movements of an antiparallel four-helix bundle formed by the G- and H-helices from each monomer of the globin domain at the dimer interface also in a ligand-dependent manner.^43^ Each H-helix of this four-helix bundle is extended and connected to the functional domain by a single long helix and is predicted to function as a signaling helix. HemAT has been proposed to utilize this ligand-dependent conformational change to transduce the structural information to the neighboring functional domain in the full-length protein.^43^ In another globin-coupled sensor, *Af*GcHK, the crystal structures of its isolated globin domain combined with a site-directed mutagenesis study also indicated that the dimer interface of the globin domain is important for the signal transduction mechanism of *Af*GcHK.^44,45^ This dimer interface was observed to also be formed by G- and H-helices, with these helices then forming a four-helix bundle with another monomer, as observed for HemAT, and each H-helix could function as a signaling helix in *Af*GcHK as well. Although the concept of a signaling helix was originally derived for sensor histidine kinases and guanylyl cyclases,^46^ this mechanism could be applied to diverse multidomain signaling proteins including bacterial globin-coupled sensors.

### Relationship between Hydrolysis of c-di-GMP Catalyzed by the HD-GYP Domain and its Dinuclear or Trinuclear Metal Center

Based on HD-GYP-domain-containing PDEs experimentally characterized so far, the presence of a Glu residue (Glu190 in GCS-HD-GYP) in the N-terminal loop of the HD-GYP domain has been indicated to be an important determinant for forming a trinuclear metal center at the active site and for proceeding to the second step of hydrolysis, i.e., the conversion of pGpG to GMP.^26^ HD-GYP-domain-containing PDEs that have a trinuclear metal center catalyze the hydrolysis of c-di-GMP to pGpG, and then of pGpG to GMP. In contrast, HD-GYP-domain-containing PDEs that have a dinuclear metal center catalyze only the first step of hydrolysis, i.e., of c-di-GMP to pGpG. According to this classification, GCS-HD-GYP belongs to the trinuclear metal center group because Glu190 is conserved compared with other relevant HD-GYP-domain-containing PDEs (Figure S2). However, in our experiments, GCS-HD-GYP catalyzed only the hydrolysis from c-di-GMP to pGpG, and no further hydrolysis from pGpG to GMP was observed (Figure 5). Our regular enzymatic assay was performed for 15 min in the presence of 1 mM MnCl_2_ (Figure 5). However, even a prolonged incubation (∼24 h) did not yield GMP (data not shown). Thus, despite the presence of Glu at position 190 and the addition of an excess amount of Mn^2+^, it is likely that the trinuclear metal center was not properly reconstituted or that only two Mn^2+^ ions, i.e., not three, are bound at the active site in the HD-GYP domain of GCS-HD-GYP.

### Other Orthologous GCS-HD-GYP

According to our bioinformatics analysis, GCS-HD-GYP is present not only in *V. fluvialis* but also in the closely related species *Vibrio furnissii*. A comparison of the sequences of *V. fluvialis* and *V. furnissii* GCS-HD-GYPs showed 77.9% identity and 87.5% similarity. This high sequence homology suggested a structural and functional similarity of these proteins. To assess the spectral and enzymatic properties of GCS-HD-GYP from more than one organism, we also carried out a preliminary characterization of *V. furnissii* GCS-HD-GYP (Figure S6). Our analyses indicated a molecular mass of 43.9 kDa for the purified *V. furnissii* GCS-HD-GYP with a C-terminal His_6_ tag (Figure S6A), a dimeric state for *V. furnissii* GCS-HD-GYP in solution (Figure S6B), and a predominantly α-helical structure for this protein (Figure S6C). Also, its spectral features were observed to be similar to those of *V. fluvialis* GCS-HD-GYP with similar gas binding capabilities (Figure S5D,E and Table 1). Although the optimal conditions for the activity of *V. furnissii* GCS-HD-GYP could be different from that for *V. fluvialis* GCS-HD-GYP, under the optimal conditions of *V. fluvialis* GCS-HD-GYP, the specific activity for the PDE activity of the Fe(III) complex of *V. furnissii* GCS-HD-GYP was 0.25 ± 0.04 μmol of c-di-GMP/min/(μmol of enzyme), that is, 10-fold less active than that of *V. fluvialis* (2.6 ± 0.1 μmol of c-di-GMP/min/(μmol of enzyme)) (Figure S6F,G). Thus, *V. furnissii* GCS-HD-GYP was concluded from our preliminary results to be less stable and active than *V. fluvialis* GCS-HD-GYP, which hampered further characterization in more detail, so we focused on *V. fluvialis* GCS-HD-GYP in this study.

### Insights into Physiological Functions of GCS-HD-GYP

**Figure 6:**
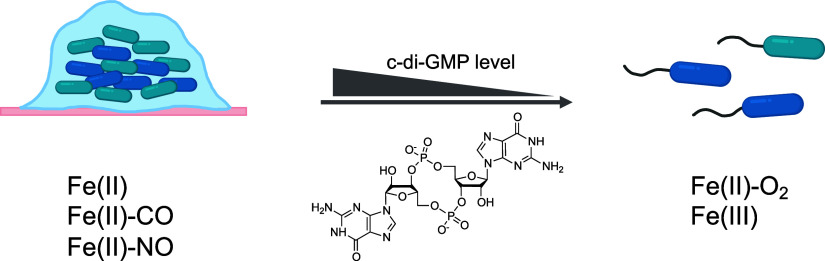
Proposed mechanism of the sensing of O_2_ and NO by GCS-HD-GYP in the human pathogen *V. fluvialis*. According to this mechanism, under normal conditions, GCS-HD-GYP is present in the active Fe(II)-O_2_ form, which hydrolyzes c-di-GMP to pGpG to increase the bacterial motility. Upon infection in a human, the host immune system induces production of NO as a bactericidal action. In response to this action, GCS-HD-GYP is switched to the inactive Fe(II)-NO form. Then, the intracellular concentration of c-di-GMP in *V. fluvialis* is increased, promoting biofilm formation to protect the bacteria from being attacked by the host immune system.

In addition to *V. fluvialis*, *V. furnissii* is also ubiquitously present in marine environments, and this presence can lead to human gastroenteritis and have extra-intestinal manifestations.^47^ Considering the recent increases in the number of diarrheal outbreaks and sporadic extraintestinal cases, both *V. fluvialis* and *V. furnissii* have been regarded as emerging human pathogens.^18^ Infections of these pathogens in humans have been mainly associated with the consumption of seafood or drinking of contaminated water.^48^ However, their mechanism of pathogenesis and survival fitness in the environment are largely unknown.^18^ Although it is difficult to determine the physiological function of GCS-HD-GYP in *V. fluvialis* and *V. furnissii* without clear phenotypic data for overexpression and/or knockout of these genes, we speculate that GCS-HD-GYP may act as a switch to regulate the bacterial lifestyles between motile planktonic and sedentary biofilm-associated lifestyles in response to changes in cellular redox status and/or gas concentration (Figure 6).

In addition, we have derived a speculative proposal for a mechanism by which these bacteria may respond to an attack by a human host cell (Figure 6). Under normal conditions, GCS-HD-GYP exists in the active Fe(II)-O_2_ state, which catalyzes the hydrolysis of c-di-GMP, promoting bacterial motility. When these bacteria infect a human, however, the host cell mounts a defense response involving the production of a lot of NO, which has bactericidal action.^49^ In a counter response, NO is sensed by GCS-HD-GYP, and GCS-HD-GYP switches from the active Fe(II)-O_2_ form to the inactive Fe(II)-NO form. This in turn causes the intracellular concentration of c-di-GMP in *V. fluvialis* to increase, which could result in the formation of a biofilm acting as a physical barrier protecting these bacteria from being attacked by the host immune system. Therefore, this system could be part of the defense mechanism of *V. fluvialis* against the host immune system upon infection. In this scenario, NO can easily replace O_2_ on the heme of GCS-HD-GYP due to the difference between their affinities for heme. In general, NO has a higher affinity for heme than does CO, which has a higher affinity than does O_2_, according to the “sliding scale rule” for selectivity among these gaseous ligands.^50^ Specifically, when the proximal ligand is a His and the distal site is apolar, dissociation constants (*K*_d_) have been shown to follow the order *K*_d,NO_ < *K*_d,CO_ < *K*_d,O_2__ with a ratio of 1:∼10^3^:∼10^6^.^50^

## Conclusions

In this study, we have identified and characterized a novel globin-coupled sensor PDE, namely, GCS-HD-GYP, from *V. fluvialis*. The Fe(III) and Fe(II)-O_2_ complexes appear to be active forms in terms of the hydrolysis of c-di-GMP, whereas the Fe(II), Fe(II)-CO, and Fe(II)-NO complexes appear to be inactive forms. Although bacterial globin-coupled sensors containing a GGDEF domain with DGC activity have been documented, no PDE containing an HD-GYP domain has been reported to date. Given the importance of c-di-GMP in infection and virulence, GCS-HD-GYP could play an important role in the ability of *V. fluvialis* to sense O_2_ and NO in the context of host–pathogen interactions. To the best of our knowledge, GCS-HD-GYP was the first globin-coupled sensor PDE to have been identified in any bacterium and is critical for the degradation of c-di-GMP in *V. fluvialis*. Our study would appear to expand our understanding of this family of globin-coupled sensors, a still-growing family among heme-based gas sensors.

## Supporting Information Available

Metal content analysis of the purified *V. fluvialis* GCS-HD-GYP; sequence analysis of the globin and HD-GYP domains of GCS-HD-GYP; predicted structural model of the full-length GCS-HD-GYP, optimization of reaction conditions for c-di-GMP hydrolysis of *V. fluvialis* GCS-HD-GYP; relationship between the heme coordination structure and its catalytic activity in GCS-HD-GYP; and characterization of *V. furnissii* GCS-HD-GYP (PDF)

## Accession Codes

*V. fluvialis* GCS-HD-GYP, UniProtKB A0A120L350; *V. furnissii* GCS-HD-GYP, UniProtKB A0A0Q2RS25.

## Abbreviations

c-di-GMP: bis(3′,5′)-cyclic dimeric guanosine monophosphate
pGpG: 5′-phosphoguanylyl-(3′,5′)-guanosine
GMP: guanosine 5′-monophosphate
DGC: diguanylate cyclase
PDE: phosphodiesterase
DEA NONOate: diethylamine NONOate
ICP-OES: inductively coupled plasma optical emission spectroscopy
CD: circular dichroism
HPLC: high-performance liquid chromatography
6cHS: 6-coordinated high-spin
6cLS: 6-coordinated low-spin
5cHS: 5-coordinated high-spin
