# Supplementary material for: Gas-Selective Catalytic Regulation by a Newly Identified Globin-Coupled Sensor Phosphodiesterase Containing an HD-GYP Domain from the Human Pathogen Vibrio fluvialis
Source: Biochemistry. 2024 Jan 24;63(4):523–32. doi: 10.1021/acs.biochem.3c00484 (PMC10882959; doi:10.1021/acs.biochem.3c00484)
Supplement: Supplementary file 1 — bi3c00484_si_001.pdf [file bi3c00484_si_001.pdf]

Supporting Information

Gas-Selective Catalytic Regulation by a Newly Identified Globin-Coupled Sensor Phosphodiesterase Containing an HD-GYP Domain from the Human Pathogen *Vibrio fluvialis*

Kenichi Kitanishi[†,*], Nao Aoyama[‡], and Motoyuki Shimonaka[†]

[†]Department of Chemistry, Faculty of Science, Tokyo University of Science, 1-3 Kagurazaka, Shinjuku-ku, Tokyo 162-8601, Japan.

[‡]Department of Chemistry, Graduate School of Science, Tokyo University of Science, 1-3 Kagurazaka, Shinjuku-ku, Tokyo 162-8601, Japan.

Corresponding Author

*Kenichi Kitanishi, Department of Chemistry, Faculty of Science, Tokyo University of Science, 1-3 Kagurazaka, Shinjuku-ku, Tokyo 162-8601, Japan, Tel.: +81-3-3260-4272 (ext. 5738), E-mail: kita24@rs.tus.ac.jp.

Table S1. Metal content analysis of the purified GCS-HD-GYP quantified by ICP-OES.

Metal	equiv
Fe	1.0
Mn	< 0.01
Co	< 0.01
Cu	< 0.01
Ni	< 0.01
Zn	< 0.01

```
                                                              49
V. fluvialis  METAFSSEPESVIRRWASYLVIDQQTLNTLREFYWVMEQNIDDILSHVYAHLSSNRETAH
V. furnissii  MEVAFGSDPGNAIRRWASYLAIDQQTVDALREYYWIMERNIDEVLAFVYDHLSSCPETAQ
HemAT         KNRIQLTNKHADVKKQLKMVRLGDAELYVLEQLQPLIQENIVNIVDAFYKNLD-HESSLM
AvGReg        ---MPGISPEQQAAEWKLLLGQFPAP--VVAQIRELATTHQSELPGYFYEQMLQDEQAML
BpeGReg       ----MKPSPEILALRWKDTCAHYSPHEWVAARN--VVTANKAALADYFYECMLADPNAAF
YddV          ----MEMYFKRMKDEWTGLVEQADPP--IRAKAAEIAVAHAHYLSIEFYRIVRIDPHAEE
AfGcHK        ----MTGVPETVFEELKRYVGWGDGDERALRSLHGAAAPHFPRLAEEFYDRILGHEGART
              .                        :   :   .*  :     :

                                                          103
V. fluvialis  FYQDEQSVKRAKAHQREHMMRYVFRG---NFGAEYYNATNRIGKTHHKLGIDFKIYSGAY
V. furnissii  FYQNEQSIVRARGHLRDHLMLYVFRG---HFGVDYYNATIRIGQTHQRLGIDFKVYSGAY
HemAT         DIINDHSSVDRLKQTLKRHIQEMFAG---VIDDEFIEKRNRIASIHLRIGLLPKWYMGAF
AvGReg        FLTHEQVKSRLHGTLRQWIVSVFSMSDDDAALQALIAQQKQIGEIHARIKIPIHLVLRGA
BpeGReg       FLSDQLVKTKLHASMQDWLESVYAAAP-TEEYERTVAFQRKVGEVHARIDIPVHLVTRGA
YddV          FLSNEQVERQLKSAMERWIINVLSAQ--VDDVERLIQIQHTVAEVHARIGIPVEIVEMGF
AfGcHK        ALVGGESQVGHLKVTMIAWLDELLGG---PWDEAYWDRRYRIGRVHVRIGLPQHYMFGAM
                                                       :.  * :: :  .    .

V. fluvialis  CIVLSQLAGVVYKVLAPEIGNVQRYMTALNRVIFMDMGLATSVYYDTAC
V. furnissii  CIVMSQLARVVYQSLAPNIDNIHRYMSALNRAIFLDLGLATAVYYDTAC
HemAT         QELLLSMI-DIYEASITNQQELLKAIKATTKILNLEQQLVLEAFQSEYN
AvGReg        RHLRERLFVLLRQRPLDPEHKLFGQR-LISETVDLAMEIMSRAFSDAYD
BpeGReg       CALIRRICELLDRDASLSAAQAAATCRYVADVTMTAVEMMCHAYSVSHD
YddV          RVLKKILYPVIFS-SDYSAAEKLQVYHFSINSIDIAMEVMTRAFTFSDS
AfGcHK        NVHRTGLARLAYERFHGDPPELERVRNALGKVLDLELAVMLHTYREDLL
                    :             :                 :   .:
```

Figure S1. Sequence analysis of the globin domain of GCS-HD-GYP. Numbering above the sequences shows residue numbers of *Vibrio fluvialis* GCS-HD-GYP. Shown are aligned amino acid sequences of the globin domain (residues 1–166) of *Vibrio fluvialis* GCS-HD-GYP (UniProtKB A0A120L350), globin domain (residues 1–166) of *Vibrio furnissii* GCS-HD-GYP (UniProtKB A0A0Q2RS25), and relevant globin-coupled sensor proteins that have been structurally characterized, namely *Bacillus subtilis* HemAT (residues 22–185) (UniProtKB O07621), *Azotobacter vinelandii* GReg (residues 1–163) (UniProtKB M9YE33), *Bordetella pertussis* GReg (residues 1–162) (UniProtKB Q7VTL8), *Escherichia coli* YddV (residues 1–160) (UniProtKB P0AA89), and *Anaeromyxobacter* sp. Fw109-5 GcHK (residues 1–162) (UniProtKB A7HD43). It is assumed that Tyr49 is located at the heme distal side, whereas His103 is the proximal axial ligand in GCS-HD-GYP, which are shown in blue and red, respectively.

```
                              194                                       226 227
V. fluvialis      KAGEFRDNETGEHIKRISRMSFELAKAAGQE--------PHWCKMIQIASPLHDVGKIGV
V. furnissii      KAGEFRDNETGEHIKRISRMSFELAKALGQP--------AHWCKMIQIASPLHDVGKIGV
PmGH              HATKFKDPETQNHIIRVGLYCEILAREAGLD--------EEDVELVKLAAPMHDIGKVGI
TM0186            MLSEYRDMETHRHTERVGWLSGRIAEEMGMS--------EVFVTEIQFAAPLHDIGKIGI
Bd1817            MNIENTDKTISHHGVTVSTLSIALAQKLGITD-------PKKTQLLTLGALLHDYGHHHS
PA4781            TLGDLRDNPRSRHLPRIERYVRLLAEHLAAQRAFADELTPEAVDLLSKSALLHDIGKVAV
Bhr-HD-GYP        TINEFHDQYTVGHESRVSNIAIDIAKKLNLD--------KDTIMAIKISALLHDIGKITV
                     .  *     *   :      :*.                   :  .: :** *:

                                       255                             281 282
V. fluvialis      PDDILLKPGKLDEQEWNVMQQHPAMGGVIIPDNK------SELIRMARRISLTHHEKWDG
V. furnissii      PDEILLKPGKLDADEWKLMQQHPAIGGDIIPDNK------SELIRMARRISLTHHEKWDG
PmGH              PDRVLLKPGKLNDEEWEIMKKHTIYGYEILKGGD------SRLLQIAADIAIEHHERWDG
TM0186            PDRILLKPGILTPEEFEIMKQHTTIGFRILSRSN------SPILQLGAEIALTHHERWDG
Bd1817            PLNLNQPLDSMSPEDLALWKKHPIEGAQKVQDKK-------HFDQTVINIIGQHEETING
PA4781            PDRVLLNPGQLDAADTALLQGHTRAGRDALASAERRLGQPSGFLRFARQIAYSHHERWDG
Bhr-HD-GYP        PQELLNKSGKISNEERDILKSHVNAGYNILKNIS--------FPWPIADIVYQHHERLNG
                  *  :    . :   :  : : *   *   :   .        :      *   *.*  :*

                                        310
V. fluvialis      SGYPAGLKGEEIPLEGRIVAICDVFDALLSTRPYKRPWSMEEVGTYLRDNRGKHFDPQLL
V. furnissii      SGYPAGLAGEEIPLEGRIVAICDVFDALLSTRPYKRAWSVAEVVAYLRENRAKHFDPLLL
PmGH              TGYPFGKKGEEISIYGRMTSISDVFDALTSDRPYKKAWDMDRTVRFFKEQKGKHFDPFLT
TM0186            SGYPRGLKEREIPISGLIVAVADSFDAMVSRRPYKNPKPLEEAFREIESLSGKLYSPEVV
Bd1817            TG-PKGLREKDMDPLAVLVSSANAMDRLITFEGVPKAEAAKKLMIDHVGKHPLQHIQHLN
PA4781            RGFPEGLAGERIPLAARIVALADRYDELTSRHAYRPPLAHAEAVLLIQAGAGSEFDPRLV
Bhr-HD-GYP        SGYPEGKHSGDILIEAKIIAVADVYESMATNRPYRQKVGHEKALEELIKGKGILYDSIVV
                   * * *     :   . : : .:  : : : .         .            .   :

V. fluvialis      DIFIENLANMQLIRCQFEDAVSPEIVAD------------------
V. furnissii      DCFLEHLDSMLHIRHQFEDAAQPETVAD------------------
PmGH              DIFLKNIDQMFSIKRELRDED-------------------------
TM0186            EAFLKLEKEITDVYRREKDEDTSHNGGRSHQSSPGEGVEGIR----
Bd1817            DILKGL----------------------------------------
PA4781            EAFVAVADAFAEVARRYADSAEALDVEMQRLEQAVAESIELTAPPA
Bhr-HD-GYP        DTLLSLVQDIN-----------------------------------
                  : :
```

Figure S2. Sequence analysis of the HD-GYP domain of GCS-HD-GYP. Numbering above the sequences shows residue numbers of *Vibrio fluvialis* GCS-HD-GYP. Shown are aligned amino acid sequences of the HD-GYP domain (residues 182–375) of *Vibrio fluvialis* GCS-HD-GYP (UniProtKB A0A120L350), HD-GYP domain (residues 182–375) of *Vibrio furnissii* GCS-HD-GYP (UniProtKB A0A0Q2RS25), and other homologous HD-GYP-domain-containing PDEs, namely *Persephonella marina* PmGH (residues 177–363) (UniProtKB C0QQ26), *Thermotoga maritima* TM0186 (residues 161–368) (UniProtKB Q9WY30), *Bdellovibrio bacteriovorus* Bd1817 (residues 138–308) (UniProtKB Q6MM30), *Pseudomonas aeruginosa* PA4781 (residues 168–393) (UniProtKB Q9HV27), and *Ferrovum*

sp. PN-J185 Bhr-HD-GYP (residues 391–565) (UniProtKB A0A149VUS3). It is assumed that His194, His226, Asp227 and Asp310 coordinate one metal (M1), whereas His255, His281, His282, Asp227 and Asp310 coordinate the other metal (M2), and Asp227 and Asp310 bridge both metal ions in GCS-HD-GYP. Putative residues involved with metal binding are shown in red, and the GYP motif is shown in blue. The Rxx(R/K) motif, shown as underlined, has been shown to be important for the specificity of the proteins for binding c-di-GMP rather than other cyclic dinucleotides. The Glu near the N-terminus of the HD-GYP domain and shown in a red box has been proposed to be important for forming the trinuclear metal center.

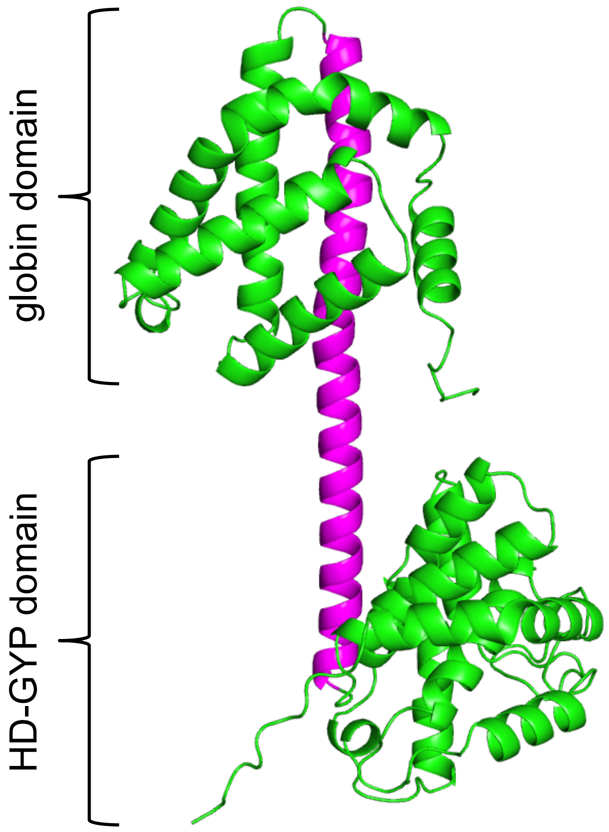

Figure S3. Structural model of the full-length GCS-HD-GYP predicted using AlphaFold. GCS-HD-GYP was indicated, according to the size-exclusion chromatography analysis in this study, to be a dimer in solution; but only a monomer is shown here for the sake of simplicity. Note that the globin domain and HD-GYP domain were modeled to be connected by one long signaling helix, which is shown in magenta.

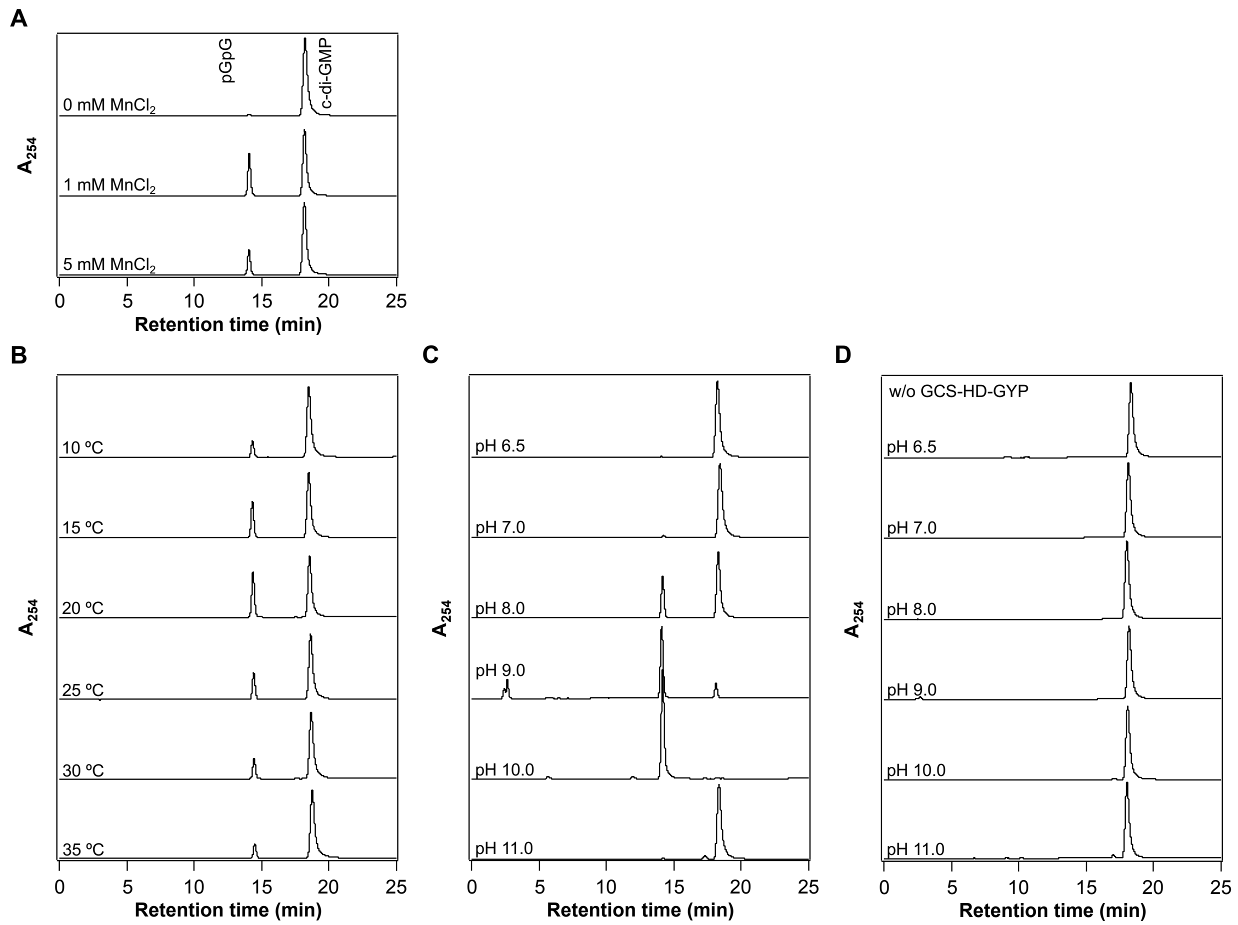

Figure S4. Optimization of reaction conditions for c-di-GMP hydrolysis catalyzed by GCS-HD-GYP. (A) Effect of the concentration of $MnCl_2$ on the catalytic activity of the Fe(III) complex of GCS-HD-GYP. HPLC profiles of reaction mixtures, each after 15 min of incubation at 20 °C, of 1 μM of the Fe(III) complex of GCS-HD-GYP in the presence of 0, 1, or 5 mM $MnCl_2$. (B) Effect of the reaction temperature on the catalytic activity of the Fe(III) complex of GCS-HD-GYP. HPLC profiles of reaction mixtures, each after 15 min of incubation at 10, 15, 20, 25, 30, or 35 °C, of 1 μM of the Fe(III) complex of GCS-HD-GYP in the presence of 1 mM $MnCl_2$. (C) Effect of the buffer pH on the catalytic activity of the Fe(III) complex of GCS-HD-GYP. HPLC profiles of reaction mixtures, each after 15 min of incubation at 20 °C, of 1 μM of the Fe(III) complex of GCS-HD-GYP in the presence of 1 mM $MnCl_2$ in a buffer with a pH value between 6.5 and 11.0. These buffers were the same as those used for experiments investigating the effect of pH on spectral data, but these buffers contained 100 mM NaCl and 1 mM $MnCl_2$. (D) Control experiment for (C) under the same conditions (20 °C, 15 min) in the presence of 1 mM $MnCl_2$ without GCS-HD-GYP.

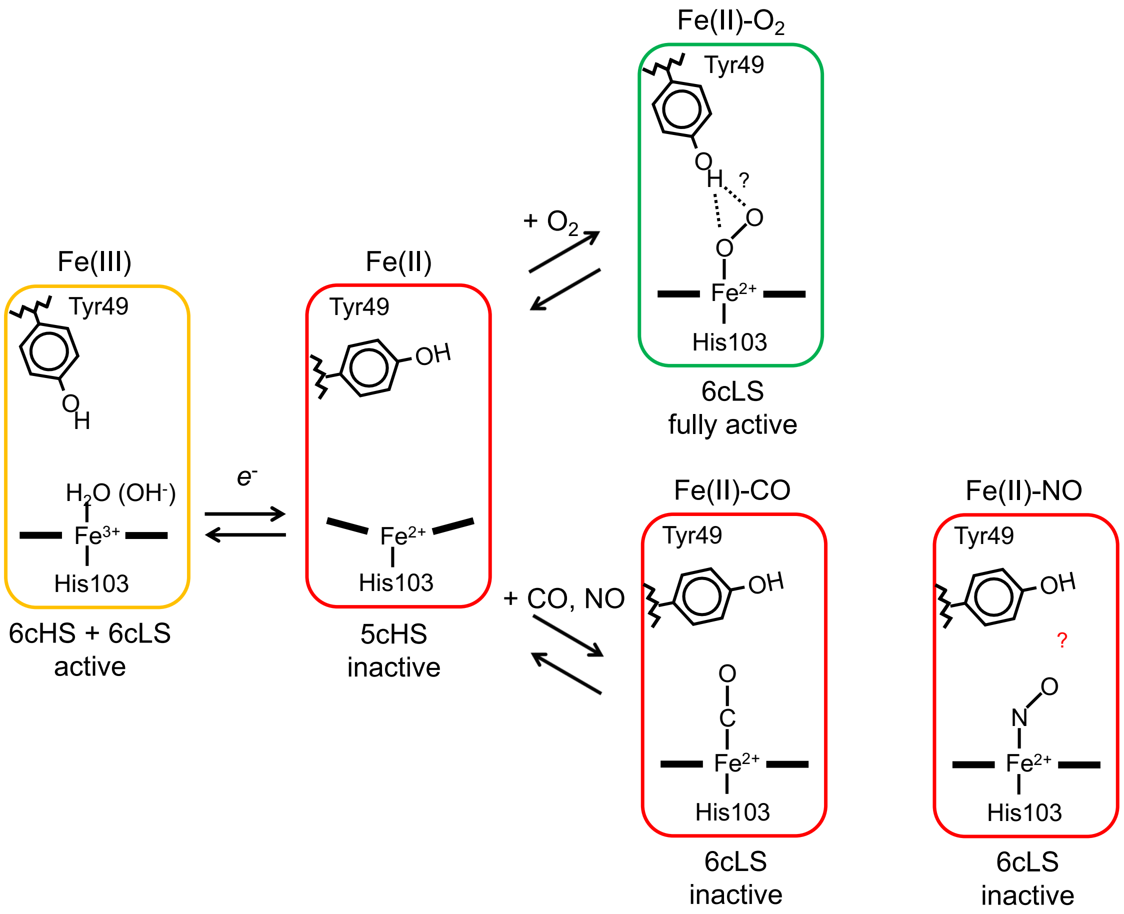

Figure S5. Schematic of the relationship between the heme coordination structure and its catalytic activity in GCS-HD-GYP.

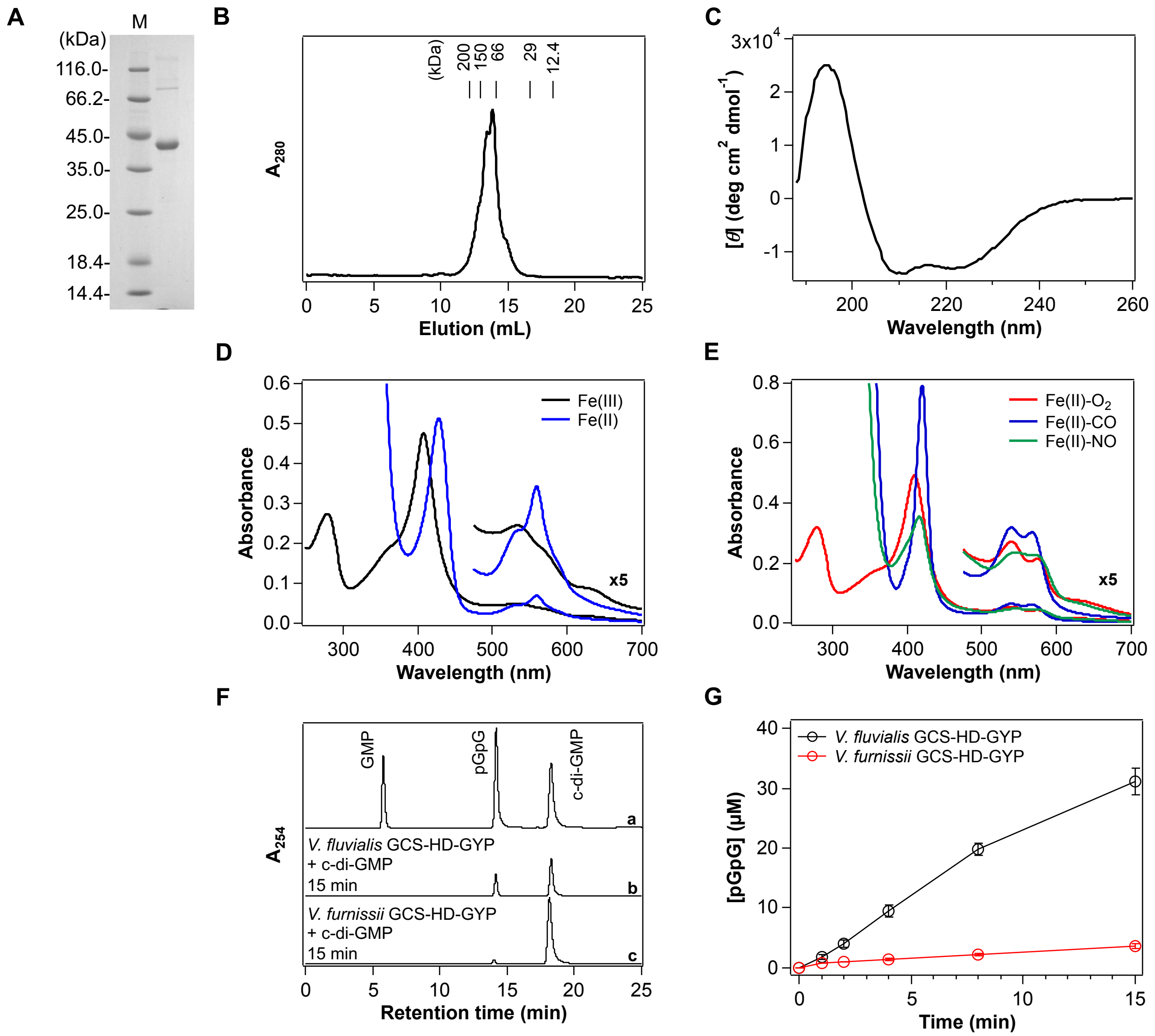

Figure S6. Characterization of *Vibrio furnissii* GCS-HD-GYP. (A) 12% SDS-PAGE of purified *Vibrio furnissii* GCS-HD-GYP. (B) Elution profile of a *Vibrio furnissii* GCS-HD-GYP sample from an analytical size-exclusion chromatography column. (C) Far-UV CD spectrum of *Vibrio furnissii* GCS-HD-GYP. (D) and (E) Absorption spectra of the indicated complexes of *Vibrio furnissii* GCS-HD-GYP. (F) and (G) Catalytic activity of *Vibrio furnissii* GCS-HD-GYP. (F) HPLC profiles of reaction mixtures, each after 15 min incubation at 20 °C, of 1 μM of the Fe(III) complex of *Vibrio furnissii* GCS-HD-GYP (trace c) and of 1 μM of the Fe(III) complex of *Vibrio fluvialis* GCS-HD-GYP (trace b). The profile of a mixture of c-di-GMP, pGpG, and GMP standards, each at a concentration of 0.1 mM, is also shown (trace a). (G) Time courses of the PDE activities of the Fe(III) complex of *Vibrio furnissii* GCS-HD-GYP (red

circles), and of the Fe(III) complex of *Vibrio fluvialis* GCS-HD-GYP (black circles). Each data point represents the mean ± S.D. of the results of at least three independent experiments.
